# DAT1-Genotype and Menstrual Cycle, but Not Hormonal Contraception, Modulate Reinforcement Learning: Preliminary Evidence

**DOI:** 10.3389/fendo.2018.00060

**Published:** 2018-02-28

**Authors:** Kristina Jakob, Hanna Ehrentreich, Sarah K. C. Holtfrerich, Luise Reimers, Esther K. Diekhof

**Affiliations:** ^1^Department of Biology, Faculty of Mathematics, Informatics and Natural Sciences, Institute of Zoology, Neuroendocrinology Unit, Universität Hamburg, Hamburg, Germany

**Keywords:** estrogen, dopamine transporter, reinforcement learning, gender, steroid hormone, hormonal contraception

## Abstract

Hormone by genotype interactions have been widely ignored by cognitive neuroscience. Yet, the dependence of cognitive performance on both baseline dopamine (DA) and current 17ß-estradiol (E2) level argues for their combined effect also in the context of reinforcement learning. Here, we assessed how the interaction between the natural rise of E2 in the late follicular phase (FP) and the 40 base-pair variable number tandem repeat polymorphism of the dopamine transporter (DAT1) affects reinforcement learning capacity. 30 women with a regular menstrual cycle performed a probabilistic feedback learning task twice during the early and late FP. In addition, 39 women, who took hormonal contraceptives (HC) to suppress natural ovulation, were tested during the “pill break” and the intake phase of HC. The present data show that DAT1-genotype may interact with transient hormonal state, but only in women with a natural menstrual cycle. We found that carriers of the 9-repeat allele (9RP) experienced a significant decrease in the ability to avoid punishment from early to late FP. Neither homozygote subjects of the 10RP allele, nor subjects from the HC group showed a change in behavior between phases. These data are consistent with neurobiological studies that found that rising E2 may reverse DA transporter function and could enhance DA efflux, which would in turn reduce punishment sensitivity particularly in subjects with a higher transporter density to begin with. Taken together, the present results, although based on a small sample, add to the growing understanding of the complex interplay between different physiological modulators of dopaminergic transmission. They may not only point out the necessity to control for hormonal state in behavioral genetic research, but may offer new starting points for studies in clinical settings.

## Introduction

The sex steroid 17ß-estradiol (E2) increases dopaminergic transmission in the reward system (1) and may enhance behavioral responses to reward and drugs of abuse [e.g., Ref. (2)]. In the animal model, E2 has been found to amplify the dopaminergic response in the striatum by (1) promoting stimulated dopamine (DA) release (3), (2) increasing DA synthesis capacity (4), and (3) decreasing the affinity of inhibitory D2-receptors (DRD2) [(5–7); but also see Ref. (8), who indicate a possible regional specificity of this effect in the striatum that might also lead to an increase]. Recent neuroimaging evidence in humans points in a similar direction. Diekhof and Ratnayake (9) showed that—similar to DA-agonistic drugs (e.g., l-DOPA)—the increase of E2 level during the late follicular phase (FP) of the menstrual cycle had the potential to enhance reward learning capacity at the expense of the ability to learn from negative feedback [see also Ref. (10)]. Previous pharmacological and behavioral genetic evidence already suggested that both intra- and inter-individual variations in dopaminergic capacity may drive differences in reward learning and punishment avoidance capacity [e.g., Ref. (11, 12)]. According to theory, the differential action of DA at two subgroups of DA receptors (DRD1 and DRD2) that are located at the direct and the indirect pathways of the basal ganglia, respectively, may determine the extent to which a person is sensitive to the rewarding as opposed to the punishing outcomes of one’s action. Phasic DA release after a rewarded action is thereby assumed to promote learning *via* long-term potentiation at the corticostriatal synapses of the direct “Go-pathway” that mainly expresses DRD1. At the same time, avoidance learning capacity is suppressed through DRD2-dependent long-term depression of the indirect “NoGo-pathway.” Conversely, when an action is followed by a dip in tonic DA, such as the one following a negative action outcome or reward omission, the NoGo-pathway is strengthened and avoidance responses are facilitated at the expense of the reward learning capacity (13). Diekhof and Ratnayake (9) found that activation of the dorsal anterior cingulate cortex (dACC) was reduced during processing of negative feedback in the late FP. Assuming that the dACC may particularly optimize action selection during avoidance learning (14), a reduced response of this brain region in a state of heightened E2 may fit well with the proposed properties of E2 in the regulation of DA release (1, 6), which should have strengthened reward learning through weakening DRD2-mediated punishment avoidance capacity.

In addition to that, there is further behavioral evidence for an inverted U-shaped relationship between baseline dopaminergic capacity, cycle-dependent changes in endogenous E2 from the early to the late FP, and reward-related decision making (15, 16) as well as working memory performance in humans (17). Smith et al. (15) assessed the influence of the COMT Met158Val polymorphism (rs4680) as a proxy of prefrontal DA on temporal discounting across the menstrual cycle. Against expectation, they found a reduction of temporal discounting (i.e., the “Now Bias”), when E2 level was increased in the late FP. Diekhof (16) used the Barratt Impulsiveness Scale (BIS) as an indirect proxy of mesolimbic DA. Using a temporal response time adaptation paradigm that is sensitive for differences in striatal DA transmission (18), Diekhof (16) assessed the ability to speed up in order to maximize reward (i.e., a measure of reward sensitivity) and compared it to the capacity to slow down for higher reward (i.e., an indicator of punishment sensitivity). Similar to Smith et al. (15), they found a paradoxical decline in the ability to speed up for higher reward from the early to the late FP, while the capacity to wait for increasing reward value improved from the low to high E2 state. Yet, when looking at inter-individual differences in hormone concentration, they also found a positive correlation between E2 concentration and an enhancement of reward sensitivity as reflected by an improved ability to speed up for higher reward. Interestingly, this correlation only existed at the lower end of the E2 distribution (i.e., in the early FP) and was further specific for subjects with low “trait impulsiveness” in the BIS, who may be characterized by a habitually low baseline DA synthesis capacity (19). In sum, these data are consistent with an inverted U-shaped relationship by showing that only subjects at the lower end of the DA distribution were affected by changes in E2, while subjects with DA levels near the point of optimality, i.e., at the center of the DA distribution, may not show such strong perturbations. Moreover, given the assumption that trait impulsiveness may to some extent mirror one’s genetic underpinnings (20–22), this finding points to a possible hormone–genotype interaction in the domain of reinforcement learning.

The present study aimed at elucidating the link between genetic predisposition and the degree to which natural variations in E2 level affect reinforcement learning. It has already been shown that genetic differences can modulate central dopaminergic transmission and cognitive functions (23). Further, individual predisposition may incline the individual to an either more reward- or punishment-sensitive learning style. For example, carriers of genetic polymorphisms, that may be associated with reduced DA autoregulation by DRD2 or an increased DA synthesis capacity, were compromised in their ability to avoid punishment in a probabilistic feedback learning task [e.g., Ref. (12, 24)]. Here, we assessed the interaction between cycle-dependent variations in E2 level and a genetic polymorphism that has been implicated in the reuptake of DA in the striatum. The 40 base-pair (40-bp) variable number tandem repeat polymorphism of the dopamine transporter (DAT1), which has been described in the 3′ untranslated region of the gene SLC6A3, has two common alleles with either 9 or 10 repeats (RP) of the 40-bp sequence The two variants may differentially affect the expression of the DAT in the striatum [(25–28), but see (29) for a null finding], and can also modulate reward-related processing (30–32), although evidence is mixed and it has not been determined, which variant may actually predispose for a higher DAT density. E2 can induce a reversal of DAT function *in vitro*, which is achieved by rapid alterations in several signaling pathways that cause efflux of DA from the DAT instead of DA reuptake (33–35). Given this evidence, the closer examination of a possible E2 by DAT1-genotype interaction and its relation to the model of the inverted U-shape of DA content and reinforcement learning capacity might add to the growing understanding of the baseline dependency of a neurocognitive function that is mediated by DA. When E2 level rises from the early to the late FP, we would predict a reversal of normal DAT function that should result in increased DA transmission in the striatum. By subdividing our sample in carriers of the 9RP allele and homozygotes of the 10RP, we should further be able to identify the genotype of the DAT1-polymorphism (9RP carriers versus subjects that are homozygote for the 10RP variant [10H]) that predisposes a person for a higher DAT density. This is because we would expect that the genetic variant that predisposes one for a higher DAT density and thus a lower DA baseline to begin with, should be more affected by the proposed E2-induced DAT reversal that is expected to happen in the high E2 state of the late FP. For one thing, in the late FP this genotype should become relatively more reward sensitive and should be less able to learn from negative feedback when heightened E2 level might induce a reversal of normal transporter function, which would promote a rise in DA level. Conversely, in the early FP when E2 is still at its nadir, we would expect the same genotype to be relatively more punishment sensitive, because the habitually higher density of DAT and thus more effective DA reuptake, that is unaffected by E2 at this point in time, would promote avoidance learning ability.

In order to test our hypothesis of a possible cycle phase by genotype interaction during reinforcement learning, our subjects performed a probabilistic feedback learning task. This task has been shown to be a sensitive measure for genetic variations in baseline DA (12, 24) and transient variations in DA concentration that were induced by pharmacological intervention [e.g., Ref. (11, 36)]. Two groups of healthy young women were genotyped for the DAT1-polymorphism. The first one had a natural menstrual cycle, while the second group took hormonal contraceptives (HC) on a regular basis. These latter women did not experience natural fluctuations of E2 level over the course of the menstrual cycle, but were influenced by synthetic ethinylestradiol and progestines, which might also affect reinforcement learning capacity. Both groups were tested twice, either in a low versus high E2 phase of the menstrual cycle (i.e., in the early and late FP) or during the “pill break” and the intake phase of HC, respectively.

## Materials and Methods

### Subjects

In total, 85 women were recruited for this study (i.e., 45 participants in the cycle group and 40 subjects in the HC group). Of these, five participants had to be excluded because of either technical problems with the response box or the test protocol, lack of compliance, or drop-out after the first test. Another subject had an exceedingly high E2 level, indicating a possible hormonal disturbance, which also led to exclusion from all further analyses. The remaining 79 healthy women [age (mean ± SEM) = 25.5 ± 0.4 years] had no current or previous psychiatric or neurological diagnosis, reported to have no history of drug abuse or gynecological problems (e.g., endometriosis) and did not have any chronic disorder related to the hormone system (e.g., diabetes, Hashimoto’s thyroiditis, PCO). Subjects were of Middle European origin as determined by the place of birth of their parents and grandparents. All subjects gave written informed consent and were paid for participation. The present study was approved by the local ethics committee (*Ethikkommission der Ärztekammer Hamburg*).

### The Cycle Group

The cycle group consisted of 39 women, who were free of any medication including hormonal contraception for at least three menstrual cycles in the past and reported to have regular menstrual cycles in the normal range of 24–36 days. Following the procedure applied by former neurobehavioral studies on the effects of E2 across the menstrual cycle (9, 15, 16), subjects from this group were only included in our final analyses if they showed an increase in E2 level from the early to the late FP. This was done because we wanted to assess the effect of rising E2 in the late FP. Ovulation is hidden in humans and the actual extent of follicular development can only be ascertained by vaginal ultrasound. Yet, a rising E2 level during the FP may be an indirect indicator of normal follicular growth. Therefore, an E2 rise from early to late FP in combination with a negative lutropin (LH) ovulation test before testing (see below) was a necessary prerequisite for the late FP test to take place, which left 31 subjects.

Since the goal of the study was to compare DAT1 9-repeat allele carriers and 10-repeat allele homozygotes in their reaction to a natural rise in E2 level and its subsequent impact on reinforcement learning capacity, the participants were also genotyped. In the present sample, 14 subjects were carriers of the 9RP allele (2 of them were homozygote for the 9RP), while the remaining 16 subjects were homozygous for the 10RP variant. There was also one person that carried the rare 11-repeat allele variant in combination with the 9RP (11/9), who was subsequently excluded, leaving the final number of 30 participants in the cycle group [age (mean ± SEM) = 27.5 ± 0.7 years; age range = 21–35 years]. About 38% of the cycle participants were currently in a relationship with a male partner and the majority considered themselves as heterosexual (two bisexual subjects).

The women were tested twice, once during the first 3 days of menstruation (early FP) and once two days before expected ovulation (late FP). The date of expected ovulation was calculated from the expected cycle length individually for each participant. For this we asked the participants to state their expected cycle length and then, upon the onset of menstruation, used the last expected cycle day to determine the optimal test day with a common counting method: for all subjects with a cycle length shorter than 28 days, we subtracted 15 days from the expected cycle end. For subjects with an expected length of 28–31 days, 16 days were subtracted, and for cycle lengths longer than 31 days, 17 days were subtracted to schedule the test in the late FP. Our subjects also determined the daily concentration of LH with a common *in vitro* urine test (One Step^®^ by AIDE Diagnostic Co., Ltd.), starting 2 days before the test date. In case of a positive result before the test day, the test was postponed to the subsequent cycle.

The test design was counterbalanced for cycle phase. Half of the participants started the test protocol in the early FP (i.e., seven carriers of the 9RP variant; eight were 10H). The remaining subjects started in the late FP (same genotype proportion).

### The HC Group

The 40 participants of the HC group were recruited when the data collection for the cycle group was almost finished. The HC group allowed us to compare the data from a phase of low hormone availability (pill break) with a phase, during which participants were under the influence of synthetic hormones (intake phase), while the impact of natural hormones was blocked. Participants from the HC group took HC for at least 12 months (mean duration of HC intake = 7.77 ± 0.61 years), but were otherwise free of medication. HC contained ethinylestradiol in the range of 0.015–0.03 mg and different amounts of progestin compounds (e.g., levonorgestrel, dienogest), which are used to suppress follicular growth and ovulation (37). Therefore, we expected no significant change in hormone level between the two test phases, except from a numerical E2 increase or a fall in E2 during the intake phase (38).

The HC group consisted of 20 carriers of the 9RP variant (one of them was homozygote) and 18 subjects were genotyped as 10H. One person in this group also had the rare 11-repeat allele variant combined with the 10RP allele (11/10) and was therefore excluded, leaving a final number of 38 subjects in the HC group [age (mean ± SEM) = 23.5 ± 0.5 years; age range = 19–32 years]. About 87% of the HC participants, i.e., almost three times as many as in the cycle group, were currently in a relationship with a male partner and the majority considered themselves as heterosexual (one bisexual subject).

The HC group had an analogous counterbalanced test design to the cycle group. Twenty participants started the test protocol in the OFF-phase (*n*_9RP_ = 10; *n*_10H_ = 10). The term “OFF-phase” refers to the so-called pill break of 7 days. The test was scheduled for the third or fourth day of the pill break. The second test phase, the “ON-phase,” required an intake of HC for at least five consecutive days. In that way, the OFF-phase resembles the early FP in terms of low E2 availability, while the ON-phase might be rather characterized by a slight influence of synthetic hormone content (in this case, the mixture of ethinyestradiol and progestin compounds contained in contraceptive medication). In order to achieve a comparable repeated test schedule to the naturally cycling women, subjects who started in the OFF-phase had the subsequent ON-test approximately 7 days later (mean ± SEM = 7 ± 1.6 days), while the other group had a gap of 18 days (mean ± SEM = 18.5 ± 0.12 days).

### Test Procedure

Participants performed a probabilistic feedback task, described in more detail by Diekhof and Ratnayake (9), which is a well-established test of reinforcement learning capacity [see also Ref. (11, 12) for description of the task]. In this task, subjects learned to associate certain stimuli with a higher probability of positive or negative feedback (i.e., a smiley or a grumpy face). During the initial learning phase (session 1), they were confronted with three fixed stimulus pairs of different hiragana and kanji symbols (i.e., pairs AB, EF, CD) from which they had to choose the one symbol that allowed them to maximize reward (positive feedback in form of a smiley) and to avoid frequent punishment (negative feedback of a grumpy face). The task goal was to receive as many smileys as possible. Unbeknownst to the participants, the reward contingencies differed between pairs and stimuli. Among all symbols, A of the pair AB was the best option (80% positive feedback upon selection), while B was the worst option (only 20% positive feedback). The reward contingencies of the remaining symbols lay in between these contingencies (i.e., pair CD: C = 70%, D = 30%; pair EF: E = 60%, F = 50%). After learning, subjects were tested in a transfer phase (session 2) to check whether they had been able to maximize positive outcome through either selection of the better option (i.e., reward learning) or by more effective avoidance of negative feedback (i.e., punishment learning). The individual learning preference cannot be dissociated from the fixed stimulus pairs of session 1, which always combined the same good and bad stimuli (e.g., A and B). This means that in session 1 the preference for the better option (e.g., for the best stimulus A in pair AB) can be equally well driven by approach of the good option A or avoidance of the bad one B. So in the transfer phase, new stimulus pairings (like AC, BD) were presented next to the original ones (AB, CD, EF). These new pairs enabled us to find out whether a subject showed a preference for the best option A (i.e., Choose A performance), which would indicate an enhanced reward learning capacity. Conversely, a participant was classified as having a high punishment learning capacity when she showed more effective avoidance of B in the new stimulus pairs (i.e., Avoid B performance) [see also Ref. (11)]. In the transfer task direct feedback was no longer provided, so that no further learning could take place. The percentage of selections of A from the old pair AB further showed the combined effect of Choose A and Avoid B performance and was thus an indicator of overall learning capacity.

Subjects were tested twice in the two phases, but with different hiragana and kanji symbols for the stimuli A, B, C, D, E, and F on each test day. Tests were scheduled in a counterbalanced sequence that was equally distributed across the two study groups and genotypes (see above).

### Genotyping

Genotyping was performed by a commercial laboratory (Bioglobe, Hamburg, Germany). DNA was extracted from buccal swabs and purified with a standard commercial extraction kit. The DAT1-polymorphism was characterized by fragment length determination of PCR products across the variable region. Assignment of genotypes was performed with the software GeneMarker v1.75 (Softgenetics). The PCR amplification procedure used the following primers: AAATAAAACTCCTTGAAACCAGC (forward primer), TGTTGTTATTGATGTGGCACG (backward primer). The distribution of genotypes was in Hardy-Weinberg equilibrium, either when calculated separately for each study group or when looking at the whole group of genotyped subjects.

### Collection and Analysis of Salivary E2

Samples of morning saliva were collected on each test day. Subjects started collection at their normal wake-up time and collected five samples in 2 ml Eppendorf tubes at regular intervals over the course of 2 h. No consumption of food or beverages other than water was allowed during this time. This collection method avoided the contamination of samples and controlled for the episodic secretion pattern of steroid hormones, thus providing a representative sample of the free E2 level on the test day. Saliva samples were frozen at −20°C for further analyses. When all samples were collected, aliquots were obtained and processed with a 17beta-Estradiol Luminescence Immunoassay (IBL International, Hamburg, Germany) following the procedure described by Diekhof and Ratnayake (9).

### Statistical Analysis

The analysis of the behavioral data was done with the software package IBM SPSS Statistics for Windows (version 22.0; IBM Corp.). Repeated-measures general linear models (GLMs) were performed on the percentage of selections of the better option made in sessions 1 and 2. The specific factors included in the GLMs are further specified in the Section “Results.” In order to examine the effect of the cycle phase during which participants entered the study on behavioral outcome in the probabilistic feedback task [see Ref. (39) for a similar procedure], we also re-calculated the GLMs as specified in the Section “Results.” For *post hoc* comparisons, either paired or independent *t*-tests were used. Pearson correlations tested for the association between individual differences in hormone level and behavioral preferences. A *p*-value smaller than 0.05 (two-tailed) was considered significant in all tests. If not otherwise indicated, we report the arithmetic mean ± SEM in the text, tables, and figures.

A more fine-grained analysis assessed the more difficult decisions to be made in session 2, which comprised the so-called “WIN-WIN trials” (i.e., pairs “AC,” “AE,” and “CE”) and the “LOSE-LOSE trials” (i.e., “BD,” “BF,” and “DF”). In the “WIN-WIN trials,” the better options A, C, and E were paired with each other and the percentage of selections of the relatively better option was measured. In the “LOSE-LOSE trials,” the worse options (B, D, F) were combined with each other and we calculated the percentage of effective avoidance of the relatively worse option. Processing of these options required a finer representation of actual stimulus value and thus indicated the individual sensitivity for more detailed value representations that subjects could have learned from the nature of positive and negative feedback in session 1 (9, 40).

## Results

### Analysis of E2 Concentration

The cycle group exhibited a significant increase in E2 level from the early to the late FP [*n* = 30; E2_early_ = 2.42 ± 0.36 pg/ml; E2_late_ = 4.35 ± 0.48 pg/ml; *t*(29) = −6.16, *p* < 0.001], while the HC group did not [*n* = 38; E2_OFF_ = 2.28 ± 0.24 pg/ml; E2_ON_ = 2.63 ± 0.25 pg/ml; *t*(37) = −1.53, *p* = 0.134]. This was also reflected by a significant group difference in the phase-related change of E2 level [Delta of E2_late–early_ cycle group = 1.93 ± 0.31 pg/ml; Delta of E2_ON–OFF_ HC group = 0.35 ± 0.23 pg/ml; *t*(56) = 4.15, *p* < 0.001].

### Behavioral Data of the Cycle Group

We tested the influence of menstrual cycle phase and thus of the natural rise of follicular E2 on behavior in the probabilistic feedback learning task separately for the learning phase (session 1) and the transfer phase (session2). We found that both overall learning (in session 1) and the capacity for learning from positive as opposed to negative feedback as demonstrated in the transfer phase were affected by the factors “*phase*” and “*DAT1-genotype”* (see Table 1 for an overview of all main effects and interactions).

**Table 1:** Main effects and interactions in the cycle group (*n* = 30).

Main effect or interaction	*F*-value	Degrees of freedom	*p*-value	Partial eta squared
**Learning phase (session 1)**				
Phase*	**6.76**	**1, 28**	**0.015**	**0.19**
Phase × DAT1-genotype*	**6.14**	**1, 28**	**0.020**	**0.18**
Pair*	**20.53**	**2, 56**	**<0.001**	**0.42**
Pair × DAT1-genotype	3.10	2, 56	0.053	0.10
Phase × pair	1.49	2, 56	0.235	0.05
Phase × pair × Dat1-genotype	1.25	2, 56	0.295	0.04
DAT1-genotype	2.25	1, 28	0.145	0.07
**Transfer phase (session 2)**				
Phase	0.49	1, 28	0.488	0.02
Phase × DAT1-genotype*	**4.89**	**1, 28**	**0.035**	**0.15**
Learning capacity*	**5.97**	**1.4, 39.7**	**0.011**	**0.18**
Learning capacity × DAT1-genotype	2.074	1.4, 39.7	0.151	0.07
Phase × learning capacity*	**8.03**	**1.4, 40.1**	**0.003**	**0.22**
Phase × learning capacity × DAT1-genotype*	**3.66**	**1.4, 40.1**	**0.049**	**0.12**
DAT1-genotype	0.13	1, 28	0.723	0.01

*Effects that are significant at p < 0.05 are highlighted in bold and are marked with an asterisk. If required violations of sphericity were corrected with Greenhouse-Geisser.

For session 1, the GLM with the two within-subject factors “*pair*” (AB, CD, EF) and “*phase*” (early FP versus late FP) and the between-subject factor “*DAT1-genotype*” (9R versus 10H) revealed significant main effects of “*phase*” [*F*(1,28) = 6.76, *p* = 0.015, partial eta squared = 0.19] and “*pair*” [*F*(2,56) = 20.53, *p* < 0.001, partial eta squared = 0.42] as well as a significant two-way interaction between “*phase*” and “*DAT1-genotype*” [*F*(1,28) = 6.14, *p* = 0.020, partial eta squared = 0.18]. All other main effects and interactions in session 1 did not reach the statistical criterion of *p* < 0.05 (see Table 1).

*Post hoc t*-tests showed that subjects exhibited enhanced learning during the early FP [overall selection of the better option across pairs: early FP = 66.69 ± 2.91%; late FP = 59.53 ± 2.45%; *t*(29) = 2.25, *p* = 0.032] and learning success followed the expected pattern of A > C > E, which was in line with the associated reward contingencies (selection of A = 70.1 ± 2.65%; selection of C = 63.46 ± 2.79%; selection of E = 55.77 ± 2.14%; significance of all *post hoc* comparisons: *p* < 0.05).

Finally, carriers of the 9RP variant showed a significant decline in the ability to select the better option during learning in the comparison of the early and late FP [*t*(13) = 3.3, *p* = 0.006], while the 10H were not [*t*(15) = 0.09, *p* = 0.927]. Moreover, during the early FP, the 9RP allele carriers were also significantly better in selecting the better option than 10H [*t*(28) = 2.56, *p* = 0.016], and this difference vanished in the late FP [*t*(28) = 0.85, *p* = 0.854] (see Table 2 for the descriptive statistics).

**Table 2:** Descriptive statistics (arithmetic mean ± SEM) of choices during reinforcement learning (session 1) and in the transfer phase (session 2), as well as current E2 level subdivided by study group, DAT1-genotype, and phase.

	Cycle group (*n* = 30)				HC group (*n* = 38)			
	9-repeat allele carriers (9/9, 9/10)		10-repeat allele homozygotes (10/10)		9-repeat allele carriers (9/9, 9/10)		10-repeat allele homozygotes (10/10)	
		
	Early FP	Late FP	Early FP	Late FP	OFF_HC_	ON_HC_	OFF_HC_	ON_HC_
**Learning phase (session 1)**								
Choose better option (%)	73.97 ± 4.47	59.04 ± 3.01	60.32 ± 3.11	59.96 ± 3.84	63.51 ± 3.36	63.65 ± 2.72	58.54 ± 2.98	58.93 ± 2.65
**Transfer phase (session 2)**								
Choose A from new stimulus pairs (%)	50.66 ± 9.10	64.94 ± 6.27	63.12 ± 5.33	75.26 ± 6.37	66.89 ± 5.45	70.39 ± 4.68	63.56 ± 5.29	65.43 ± 4.39
Avoid B in new stimulus pairs (%)	78.32 ± 5.66	52.27 ± 6.70	62.91 ± 4.79	60.96 ± 5.98	68.28 ± 4.72	70.98 ± 5.46	65.56 ± 4.13	59.83 ± 3.26
E2 level (pg/ml)	2.38 ± 0.56	4.24 ± 0.56	2.45 ± 0.48	4.45 ± 0.76	2.27 ± 0.34	2.49 ± 0.31	2.28 ± 0.34	2.78 ± 0.42

For session 2, the GLM with the two within-subject factors “*learning capacity*” (Choose A, Avoid B, old pair AB) and “*phase*” (early versus late FP) and the between-subject factor “*DAT1-genotype”* (9RP versus 10H) revealed a significant main effect of “*learning capacit*y” [*F*(1.42,39.69) = 5.97, *p* = 0.011, partial eta squared = 0.18], the two-way interactions of “*phase*” and “*DAT1-genotype*” [*F*(1,28) = 4.88, *p* = 0.035, partial eta squared = 0.15], and of “*phase*” and “*learning capacity*” [*F*(1.43,40.08) = 8.032, *p* = 0.003, partial eta squared = 0.22]. The respective *post hoc t*-tests showed that the participants of the cycle group were better at choosing symbol A in the old stimulus pair AB compared to the new pairings (*p* < 0.05 in all *post hoc* comparisons). They were also better at choosing the best option A from all other options in the new stimulus pairs when being in the high E2 state [Choose A performance: early FP = 57.3 ± 5.14%; late FP = 70.44 ± 4.51%; *t*(29) = −2.05; *p* = 0.049], and became significantly worse during avoidance of B in the new stimulus pairs [Avoid B performance: early FP = 70.1 ± 3.88%; late FP = 56.90 ± 4.46%; *t*(29) = 2.39; *p* = 0.024].

**Figure 1:**
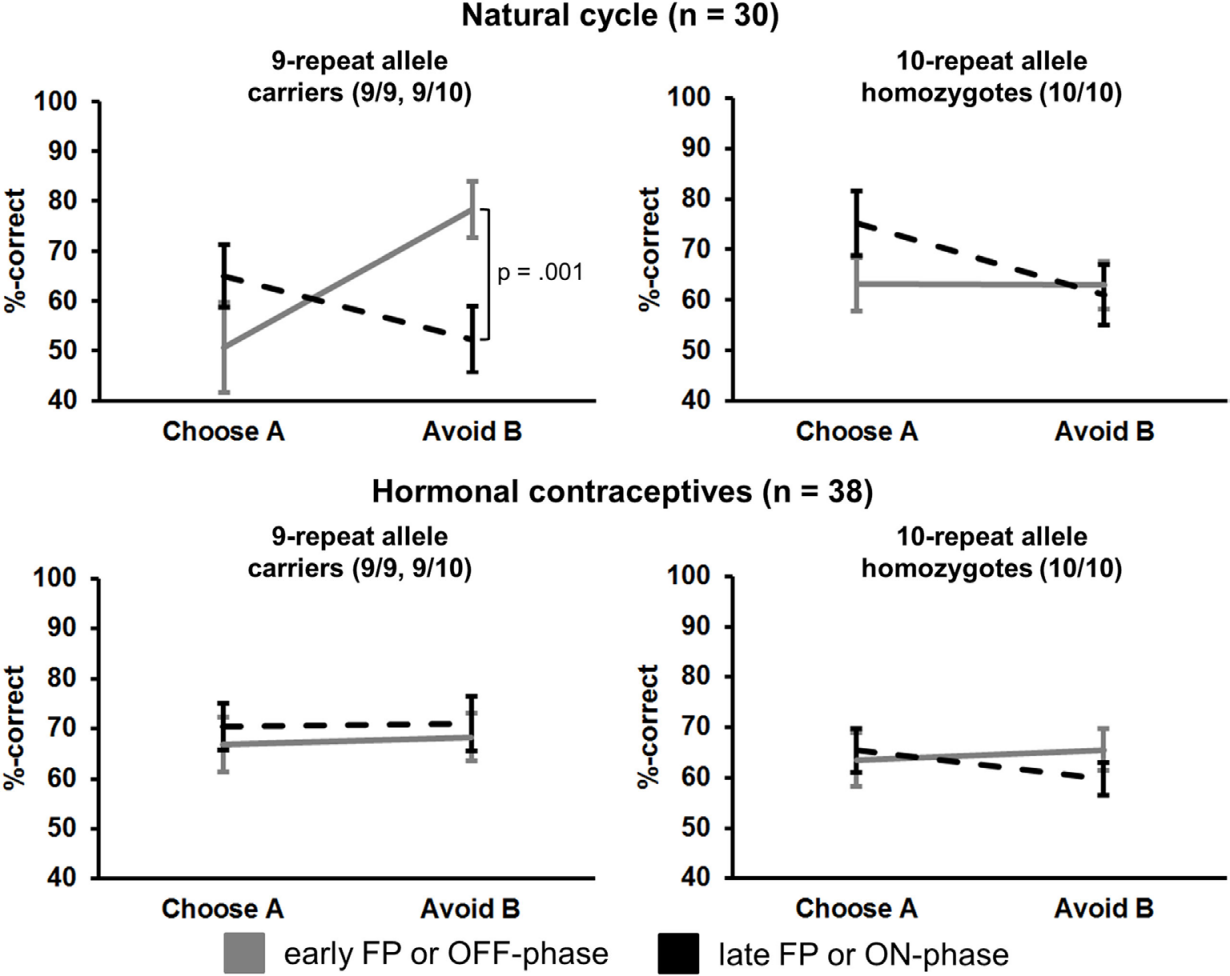
Interaction of “*phase*” × “*preference*” × “*study group*” × “*DAT1-genotype”* in the transfer phase (session 2). Carriers of the 9RP in the cycle group experienced a significant decline in the ability to avoid punishment from the early to the late FP (top left graph). Neither the 10H from the cycle group nor 9RP carriers or 10H from the HC group showed this difference between phases.

**Figure 2:**
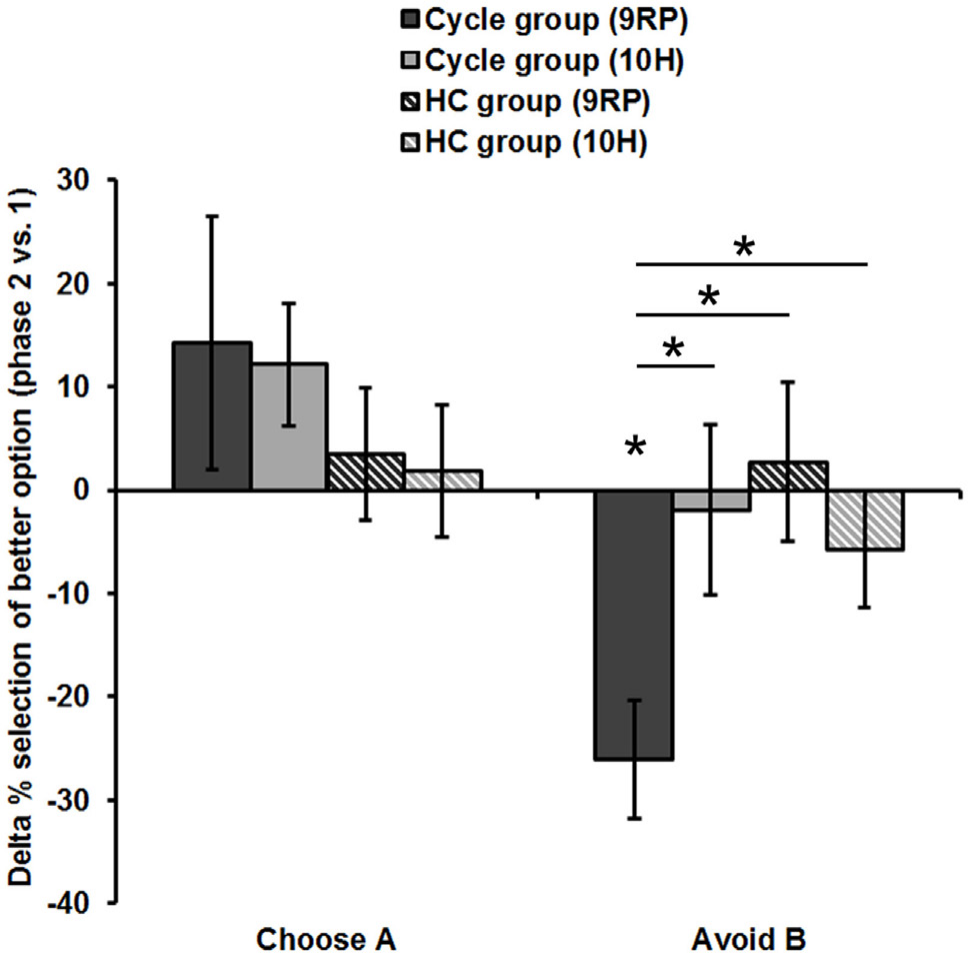
Difference in reinforcement learning capacity between phases (session 2). Displayed is the Delta of the late minus early FP and of the ON- minus OFF-phase, respectively. Only the 9RP genotype showed a significant phase-related change in punishment avoidance capacity. Significant differences (*p* < 0.05, two-tailed) from zero and between genotypes and groups, respectively, are marked with an asterisk. Phase 1, early FP or OFF-phase; phase 2, late FP or ON-phase.

Most notably, we also found the hypothesized three-way interaction of “*phase*” by “*learning capacity*” by “*DAT1-genotype*” [*F*(1.43,40.08) = 3.66, *p* = 0.049, partial eta squared = 0.12]. Carriers of the 9RP allele in the cycle group experienced a significant decline in the ability to avoid punishment (i.e., Avoid B performance) from the early to the late FP [*t*(13) = 4.53, *p* = 0.001], while the 10H from the cycle group did not show this difference between phases [*t*(15) = 0.24, *p* = 0.816] (Table 2; Figure 1). Further, in the direct comparison of genotypes, it became obvious that the difference in learning capacity was not only evident in the Delta between early and late FP, which was significantly bigger in the carriers of the 9RP [Delta Avoid B: *t*(28) = −2.34; *p* = 0.024; Delta Old pair AB: *t*(28) = −2.87; *p* = 0.008], but there was also a significant group difference in the early FP with the 9RP variant carriers outperforming the 10H in avoidance learning [Avoid B performance: *t*(28) = 2.10, *p* = 0.045] and in the Old pair AB [*t*(28) = 2.32, *p* = 0.024] (see also Figure 2). These findings suggested that the original two-way interaction of “*phase*” by “*learning capacity*” was most likely driven by the pronounced behavioral change observed in the 9RP allele carriers.

Based on this assumption a more detailed analysis of the value representations in “WIN-WIN-” and “LOSE-LOSE-trials” was performed with the 9RP variant carriers and 10H. In line with the observation of a reduced punishment sensitivity in the late as opposed to the early FP in 9RP allele carriers, we found a reduced ability to avoid the least rewarded symbols in the more difficult “LOSE-LOSE trials” for this genotype [avoidance of worst option from difficult pairs: early FP = 82.48 ± 4.14%; late FP = 64.33 ± 4.30%; *t*(13) = 3.95, *p* = 0.002], but not for the 10H [early FP = 71.76 ± 4.36%; late FP = 72.06 ± 4.14%; *t*(15) = −0.43, *p* = 0.966]. No significant difference emerged in the “WIN-WIN trials” for the 9RP carriers [choice of better option from difficult pairs: early FP = 28.66 ± 6.31%; late FP = 39.74 ± 5.81%; *t*(13) = −1.17, *p* = 0.263], nor the 10H [early FP = 40.52 ± 3.96%; late FP = 49.71 ± 4.33%; *t*(13) = −2.09, *p* = 0.055].

### Behavioral Data of the HC Group

In order to find out whether the intake of HC (i.e., synthetic estrogens) may evoke similar changes in reinforcement learning capacity as natural fluctuations in E2 during the FP, we tested this second group of subjects once during the pill break and once during the intake phase of HC. In contrast to the cycle group, we were unable to document a phase-related change in learning performance in session 1 nor was there an effect of HC intake on Choose A and Avoid B performance in session 2. Further, genotype also did not affect performance, whether considered alone or in the interaction with test phase (see Table 3).

**Table 3:** Main effects and interactions in the HC group (*n* = 39).

Main effect or interaction	*F*-value	Degrees of freedom	*p*-value	Partial eta squared
**Learning phase (session 1)**				
Phase	0.02	1, 36	0.894	<0.01
Phase × DAT1-genotype	0.004	1, 36	0.950	<0.01
Pair*	**24.34**	**2, 72**	**<0.001**	**0.40**
Pair × DAT1-genotype	1.27	2, 72	0.288	0.03
Phase × pair	0.50	2, 72	0.609	0.01
Phase × pair × Dat1-genotype	0.18	2, 72	0.833	<0.01
DAT1-genotype	1,73	1, 36	0.197	0.05
**Transfer phase (session 2)**				
Phase	0.01	1, 36	0.944	<0.01
Phase × DAT1-genotype	0.59	1, 36	0.447	0.02
Learning capacity*	**9.84**	**1.7, 60.6**	**<0.001**	**0.22**
Learning capacity × DAT1-genotype	0.29	1.7, 60.6	0.713	0.01
Phase × learning capacity	0.29	1.6, 57.1	0.697	0.01
Phase × learning capacity × DAT1-genotype	0.18	1.6, 57.1	0.788	0.01
DAT1-genotype	2.72	1, 36	0.108	0.07

*Effects that are significant at p < 0.05 are highlighted in bold and are marked with an asterisk. If required violations of sphericity were corrected with Greenhouse-Geisser.

### Group Comparison

For the group comparison (cycle versus HC group), we used the phase-related Deltas of learning performance in the two groups (i.e., Delta of “late–early FP” and Delta of “ON–OFF-phase,” respectively). We thereby intended to assess the impact of the between-subject factors “*study group*” (cycle versus HC group) and “*DAT1-genotype*” (9RP allele carriers versus 10H) on the within-subject factor “*Delta learning capacity*,” which contained the three Deltas of Choose A, Avoid B, and Old pair AB performance from session 2. The GLM revealed a trend-wise three-way interaction of “*Delta learning capacity*” by “*study group*,” and “*DAT1-genotype*,” [*F*(1.52, 97.23) = 2.93, *p* = 0.072, partial eta squared = 0.04], a significant two-way interaction of “*Delta learning capacity*” and “*study group*” [*F*(1.52, 97.23) = 3.62, *p* = 0.042, partial eta squared = 0.05] and a significant main effect of “*Delta learning capacity*” [*F*(1.52, 97.23) = 6.68, *p* = 0.004, partial eta squared = 0.10].

Accordingly, 9RP allele carriers of the cycle group showed a stronger phase-related decline in avoidance learning from the low to the high E2 phase [Delta of Avoid B performance in 9RP: cycle group = −26.06 ± 5.74%; HC group = 2.70 ± 7.7%; *t*(32) = −2.76, *p* = 0.005; Delta of Old Pair AB performance in 9RP: cycle group = −26.22 ± 6.31%; HC group = 2.15 ± 7.7%; *t*(32) = −2.7, *p* = 0.011] (see also Figure 2). Further, in the late FP, 9RP allele carriers of the cycle group were also more compromised in the ability to avoid negative feedback and to choose symbol A from the old stimulus pairs, when being compared to the same genotype in the HC group and the ON-phase [Avoid B performance in 9RP: *t*(32) = −2.18, *p* = 0.037; Old Pair AB performance in 9RP: *t*(32) = −2.31, *p* = 0.027; see also Table 2 for descriptive statistics].

In contrast, there were no phase-related differences to be found between the 10RP allele homozygotes from the cycle and the HC group. The two *post hoc t*-tests for the main effect and the two-way interaction yielded only weak results. For one thing, in the comparison of the groups there was a non-significant, numerical difference between the negative Delta of Avoid B performance [Cycle group = −13.2 ± 5.53%; HC group = −1.29 ± 4.85%, *t*(66) = −1.62, *p* = 0.11] and the positive Delta of Choose A performance [Cycle group = 13.14 ± 6.41%; HC group = 2.73 ± 4.46%, *t*(67) = 1.37, *p* = 0.18], which were both less pronounced in the HC group. Further, when considering all subjects together, there was a significant difference between the mean Delta of Choose A performance (7.32 ± 3.79%) and Avoid B performance (−6.55 ± 3.69%) [*t*(67) = 2.46, *p* = 0.016]. Yet, we presume that any group-related differences had their origin in the strong decline of avoidance learning capacity from the early to the late FP that was rather specific for 9RP variant carriers of the cycle group.

### Effect of Initial Test Phase

We also wanted to examine whether the test order may have had a significant influence on reinforcement learning capacity as demonstrated by previous studies. Following the procedure described by Wallen and Rupp (39), we performed two GLMs on the data of the initial test day, when subjects were still naïve with regard to performing the probabilistic feedback task.

For session 1, the GLM included the within-subject factor “*pair*” (AB, CD, EF) and the between-subject factors “*test phase*” (early FP, late FP, OFF-phase, and ON-phase) and “*DAT1-genotype”* (9RP versus 10H). No significant effects emerged. Only the interaction between “*pair*” and “*DAT1-genotype”* reached statistical trend level [*F*(2, 120) = 2.54, *p* = 0.083, partial eta squared = 0.04].

For the transfer phase, the within-subject factor “*learning capacity*” (Choose A, Avoid B, old pair AB) and the two between-subject factors “*test phase*” (early FP, late FP, OFF-phase, and ON-phase) and “*DAT1-genotype”* (9RP versus 10H) we included in the GLM. Here, we found a significant main effect of “*learning capacity*” [*F*(1.43, 85.42) = 13.84, *p* < 0.001, partial eta squared = 0.19] as well as a significant two-way interaction of “*learning capacity*” and “*test phase*” [*F*(4.27, 85.42) = 2.66, *p* = 0.018, partial eta squared = 0.12] and a significant three-way interaction that also included the factor “*DAT1-genotype”* [*F*(4.27, 85.42) = 2.93, *p* = 0.023, partial eta squared = 0.13]. The *post hoc t*-tests confirmed that only the 9RP allele carriers were significantly worse in the avoidance of negative feedback in the LF as opposed to the EF [9RP—Avoid B performance: EF = 87.82% ± 5.59%; LF = 39.56% ± 8.37%; *t*(12) = 4.80, *p* < 0.001], while the 10H were not [10H—Avoid B performance: EF = 64.24% ± 7.47%; LF = 67.28% ± 8.36%; *t*(12) = −0.27, *p* = 0.790]. Moreover, performance was significantly different between genotypes in both the EF [*t*(13) = −2.46, *p* = 0.028] and the LF [*t*(13) = 2.33, *p* = 0.036].

In addition, the women with a natural cycle and the 9RP variant also showed a significant performance decline in the LF (Avoid B = 39.56% ± 8.37%) in comparison to the ON-phase (Avoid B = 79.59 ± 6.51%) [*t*(15) = −3.83, *p* = 0.002]. In contrast, the comparisons of the EF and the OFF-phase in the same genotype and those in the 10H did not yield significant differences in avoidance learning between study groups and phases.

### Pearson Correlations between Phase-Dependent E2 Concentration and Performance in the Transfer Phase

To better understand the association between the individual variation in E2 concentration within the different groups and phases and reinforcement learning capacity, we also explored the respective hormone-behavior correlations in the transfer phase [see Ref. (16) for a similar approach]. First, we found a positive correlation between early follicular E2 level and reward sensitivity (i.e., Choose A performance) across genotypes in the cycle group (*n* = 30, *r* = 0.39, *p* = 0.034, two-tailed). No significant correlation emerged in the late FP or in the HC group.

Second, when subdividing the two samples by genotype, only the 9RP carriers of the cycle group exhibited a trend-wise positive correlation in the early FP (*n* = 14, *r* = 0.50, *p* = 0.07, two-tailed), suggesting that this genotype may have primarily driven the positive correlation in the complete cycle group. In contrast, the carriers of the 9RP from the HC group showed the reverse correlation in the OFF-phase (*n* = 20, *r* = −0.49, *p* = 0.027, two-tailed), and the two correlation coefficients were also significantly different (Fisher *r*-to-*z* transformation: *z* = 2.8, *p* = 0.0051, two-tailed).

## Discussion

Interactions of genotype and hormonal state have been widely ignored by cognitive neuroscience [but see Ref. (15, 17)]. The present study is the first to systematically assess how the natural rise of E2 level in the late FP may interact with DAT1-genotype during reinforcement learning. Our data show that the effect of DAT1-genotype on reinforcement learning may indeed interact with transient hormonal state, but only in women with a natural menstrual cycle. Notably, we found that carriers of the 9RP variant experienced a significant decrease in the ability to avoid punishment from the early to the late FP. No such plasticity emerged in the HC group or in the 10H from either group.

### DAT1-Genotype and Avoidance Learning Capacity

Our main finding of a reduced punishment learning capacity in 9RP allele carriers during the high E2 state (i.e., the late FP) conforms to the idea that physiological E2 may act as an endogenous DA-agonist that amplifies central dopaminergic transmission (1, 34, 35). Previous studies have already shown that synthetic drugs that increase striatal DA level may reduce the ability to avoid punishment and may in turn increase reward learning ability (11, 40). A similar effect has also been documented for changes in endogenous hormone level over the course of the menstrual cycle (9, 10, 16). Finally, behavioral genetic studies showed that the sensitivity for negative feedback may be reduced in carriers of polymorphisms associated with a higher DA synthesis capacity and reduced DRD2 density [e.g., the minor A+ allele of the Taq1A polymorphism (12, 24)]. The present study is the first to demonstrate the interactive effect of a state of physiologically increased E2 level (i.e., the late FP) and a genetic polymorphism that might be related to lower striatal DA transmission on intra-individual variations in reinforcement learning capacity. Most previous neuroimaging studies on the DAT1-polymorphism used mixed sex samples and failed to control for menstrual cycle phase in women. It currently remains unclear whether in comparison to the 10RP allele the 9RP variant of the DAT1-polymorphism is associated with a lower (25) or higher density of striatal DAT (26–28), if there is an association with DAT expression at all (29). Behavioral evidence is also mixed, with some studies suggesting that homozygosity of the 10RP allele may enhance reward-related responses [e.g., Ref. (32)], while others found evidence for the opposite with increased activation in 9RP allele carriers [e.g., (30, 31)]. The present data may provide preliminary evidence for the assumption that the 9RP variant may be related to a higher DAT density. If the state of enhanced E2 indeed induced a reversal of normal DAT function that promoted increased DA efflux as suggested by Watson et al. (35), then subjects with the genetic predisposition for a higher density of the DAT should be more strongly affected by this effect of rising E2 in the late FP. This is because the strongest E2-induced DA efflux through the DAT might be expected in individuals carrying the highest density of transporters, which should then also lead to a pronounced behavioral change from the early to the late FP. In addition to that, the same genotype should also have a higher punishment avoidance capacity in comparison to the other variant at cycle onset, when E2 level is at its nadir. Carriers of the 9RP allele of the cycle group fulfilled these two prerequisites, when being compared to homozygotes of the 10RP variant (see Figure 1; Table 2). Following this logic, the present results may also conform to the model of the inverted U-shape relationship between behavioral performance, baseline DA level, and DA-agonistic substances (41). More specifically, according to this model only subjects with a low baseline DA concentration, who according to our data might be the carriers of the 9RP allele, should be significantly influenced by a physiological rise in E2 level [see also Ref. (16)]. In contrast, the E2 rise in the late FP should have no or only a small effect on punishment learning capacity in subjects with a supposedly higher baseline DA capacity to begin with (as demonstrated here for the 10H).

However, the present observations do not conform to the results of a recent behavioral genetic study that assessed social reward learning capacity in healthy young men. Eisenegger et al. (42) used pharmacological intervention with l-DOPA, which transiently enhances striatal DA levels and assessed the interaction with DAT1-genotype (comparison of carriers of the 9RP allele versus 10H) on learning from economic interactions with either prosocial or antisocial partners. They found an increase of social learning success resulting in enhanced interactions with prosocial partners and higher pay-off under l-DOPA treatment of male 10H. In contrast, male 9RP allele carriers appeared to be impaired in learning from prosocial interactions following treatment with l-DOPA. Yet, there are several differences between the two studies that may explain the divergent findings. First, Eisenegger et al. (42) used a between-subjects design, in which both the pharmacological intervention (l-DOPA versus placebo) and the pairing with a prosocial versus antisocial partner varied between participants. This did not only result in relatively small samples for comparison (e.g., of the group confronted with a prosocial partner, only 16 of the 43 male 9RP allele carriers were treated with l-DOPA), but may have also increased the influence of inter-individual variance. For example, Eisenegger et al. (42) did not control for other genetic polymorphisms in the DA system nor in related neurotransmitter systems (e.g., the serotonin system) that could have significantly affected reinforcement learning capacity or social cognition thus contributing to group differences independent of the pharmacological intervention. When we assessed whether the effects identified in the present study were already evident during the initial, naïve test day, we were also required to use a between-subjects approach. Yet, our finding from the initial test day was in line with the finding that emerged in the within-subject design, which allowed us to rule out these potential confounds related to intersubject variation. Further, the l-DOPA treatment in healthy young men may have induced a supraphysiological DA level. The risk of dopaminergic overstimulation is always immanent when using an effective agent like l-DOPA in healthy young adults. It has already been demonstrated that the effects of supraphysiological stimulation on the DA system are not necessarily comparable to those achieved by stimulation in the physiological range and could even reverse the expected behavioral effects (3), which might explain the discrepant findings of Eisenegger et al. (42). Finally, and most importantly, Eisenegger et al. (42) assessed young men, while the present study was restricted to a female sample. Sex differences in the neural correlates of reward processing have repeatedly been demonstrated [e.g., Ref. (43)]. Further, one may speculate that a high endogenous testosterone concentration in men might have a similar effect on DAT function as rising E2 in women. The DA-agonistic effects of testosterone have repeatedly been documented (44), and testosterone may exert some of its central effect through conversion to E2. It is therefore possible that the DA response of young men, who were at their point of peak fertility, may have also been influenced by their current testosterone level, which may have further contributed to inter-individual differences between subjects. Again, this renders the risk of overstimulation by l-DOPA even more likely and might further explain discrepant findings between the two studies.

It has recently been argued that test order effects may render the interpretation of the results from previous studies on the menstrual cycle difficult (39, 45). For this reason, we analyzed the data from the initial test day separately and found that the results from the analysis of the influence of cycle phase during this naïve test replicated the effects of the repeated-measures design. For this reason, we are confident to assume that the effects identified in this preliminary study could indeed be a consequence of the hypothesized cycle phase by genotype interaction.

### Differences between the Cycle and the HC Group

Another important finding of the present study was that the decrease in avoidance learning capacity was restricted to the women from the cycle group who experienced the effect of a natural rise in E2 in the late FP. The HC group showed no behavioral variations between the pill break and the intake phase. Since the intake of HC suppressed the physiological rise in E2 to such an extent that only a slight numerical increase of E2 concentration remained at the group level (Table 2), the lack of effect of natural E2 on reinforcement learning ability was not unexpected. Yet, our data also suggest that the women of the HC group remained unaffected by the intake of synthetic hormones. This was in so far surprising as more general differences have been hypothesized to exist between women with a natural menstrual cycle and those that take HC on a regular basis [e.g., Ref. (46)]. The HC sample consisted of women that took HC for at least 1 year, with an average intake duration of more than 7 years. This might have induced profound adaptations to the constant hormonal treatment, such as a compensatory reorganization of neuroanatomy or function that has been demonstrated elsewhere and may also promote behavioral differences (37). When considered as deflections from homeostasis, compensatory long-term adaptations should become particularly evident when the pharmacological agent is with-held (i.e., during the pill break) and may then show up as deflections from homeostasis. Such a mechanism might be comparable to the long-term drug effects on the DA system in substance abuse disorders (47). Yet, the HC subjects from the present study remained quite stable across phases when considering the mean performance in the probabilistic feedback learning task (Table 2), which could indicate that long-term HC might have rendered the brain rather unresponsive to the change induced by a short OFF-period. Only the observation of a negative correlation between E2 level and reward sensitivity in the OFF-phase, which contrasted the positive correlation documented in the cycle group, may hint us to the possibility of compensatory long-term adaptations, a speculation, which however needs to be replicated in a bigger sample.

Only a limited number of studies have so far assessed the influence of HC on brain function and anatomy. Bonenberger et al. (48) examined the influence of HC in the context of reward processing in a modified version of the monetary incentive delay task. They demonstrated that the regular intake of HC may slightly alter activation in the anterior insula during reward expectation, but not in the striatum nor in other regions of the mesolimbic DA system. In addition, another two studies suggested HC-related changes in brain regions that are important for various fundamental aspects of cognition (46), which may in part depend on the subtype of HC used [for example in relation to face recognition performance (49)]. In the present sample, 17 women (*n*_9RP_ = 9) used HC containing androgenic progestins, while the remaining 21 subjects (*n*_9RP_ = 11) took HC with anti-androgenic progestins. Exploratory *t*-tests revealed no significant behavioral differences between the HC subtypes at *p* < 0.05 (two-tailed), also not when accounting for the influence of genotype and pill phase. Yet, the present sample was quite small. Since the DA-agonistic properties of androgens have already been demonstrated (44), it might be valuable to readdress the potential impact of HC subtype (androgenic versus anti-androgenic progestins) on reinforcement learning in a bigger sample.

Alternatively, it might also be possible that any changes in reinforcement learning capacity from the OFF- to the ON-phase were masked by a rise in progestin content that accompanied the synthetic estrogen administration. If that was the case, the DA-antagonistic properties of progesterone and its metabolites [e.g., Ref. (50–52); see also Ref. (9)] would have neutralized any E2-related effect on dopaminergic transmission in the ON-phase. Since we did not measure salivary progesterone level, we are unable to rule out this latter possibility.

Finally, other group differences, like the fact that most HC subjects were in a committed relationship, while less then half of the participants from the cycle group indicated to have a partner, could have also contributed to differences in the underlying neurofunctional structure, since partnership has been shown to affect the hormone system, which could indirectly influence brain physiology [e.g., Ref. (53)].

### No Evidence of a Cycle Phase by Genotype Interaction in the Context of Reward Learning Capacity—A Possible Relation to Tonic DA?

But why did the 9RP allele carriers become compromised in avoidance learning ability without experiencing an increase in reward sensitivity from the early to the late FP? The functional opponency of reward and punishment learning capacity has been demonstrated repeatedly and can be induced by both variations in central DA transmission [high versus low baseline DA, respectively, e.g., Ref. (11, 13)] and endogenous E2 level (9, 10, 16). The present data indicated a slight rise of the “Choose A” performance from the early to the late FP in both 9RP variant carriers and in 10H in the transfer phase (Table 2; Figure 1), which, against the background of a decline in “Avoid B” performance, would be in line with the assumption of functionally opponent processes. Yet this increase was not significant. This latter observation may fit with previous evidence suggesting that if tonic DA level is low, the transient upregulation of the phasic DA response can still be observed, which may preserve a normal responsiveness to reward (54). Therefore, even relatively lower levels of DA (e.g., those expected during the early FP) may be sufficient for effective learning from reward through a positive reward prediction error (55). This might have been one explanation for the observation that the rise in E2 level in the late FP had no further enhancing effect on reward learning capacity in the probabilistic feedback task. Diekhof and Ratnayake (9) documented a similar finding, when comparing the late FP and the luteal phase of the menstrual cycle and also found no evidence for a change in striatal processing of positive feedback. In contrast to that, the ability to avoid negative feedback appeared to be more sensitive to variations in tonic DA level. In order to realize effective learning from the negative outcome of an action, a significant depression of tonic DA is required (55, 56). Moreover, the dip in dopaminergic tone has to be strong enough for engaging the respective corticostriatal connections of the indirect NoGo-pathway of the basal ganglia to realize effective punishment learning in the probabilistic feedback task (13). One may assume that in the E2-dominated state of the late FP and the supposedly higher DA content in the striatum, the suppression of dopaminergic tone following a negative feedback would become more difficult. This would decrease the signal-to-noise ratio and thus render the negative prediction error signal less likely, which would ultimately result in a significant decline in punishment learning capacity. Diekhof and Ratnayake (9) demonstrated a significant decline in “Avoid B” performance in the late FP, which was also accompanied by a reduced activation of the dACC by negative feedback. In the present study, the highest sensitivity for more detailed value representations in “LOSE-LOSE trials” was evident in the 9RP allele carriers in the early FP, and this sensitivity declined by 18% when E2 level rose in the late FP. Moreover, carriers of the 9RP variant also showed a cycle phase-related difference in learning performance in session 1. Subjects became particularly worse when learning from pair AB [early FP = 79.3 ± 4.6%; late FP = 62.8 ± 4.8%; *t*(13) = 2.77, *p* = 0.016]. This suggests a reduced ability to identify the least rewarded option already in the fixed stimulus pairs.

Nevertheless, the prediction error theory of dopamine may provide only one possible explanation for the current and previous findings [e.g., Ref. (9)] observed in the probabilistic feedback task. Alternatively, variations in the motivation to act or to engage in goal-directed action might have equally well contributed to the observed differences between cycle phase-related variations in punishment and reward learning capacity [see Ref. (57) for review].

### Limitations

First, in this initial study, we tested a relatively small sample of women [in the range of previous behavioral genetic studies; e.g., Ref. (17)] and looked at a single polymorphism that may affect DAT density in the striatum. For these reasons, the present results can only be considered as preliminary and require further replication.

Second, the cycle phase by genotype interaction had a relatively small influence on variations in reinforcement learning capacity, as indicated by the small effect size of the interaction when the factor “*study group*” (HC versus cycle group) was also taken into account (partial eta squared < 0.10). However, the present study used a rather conservative approach by comparing phase-related changes in both a cycle group and a sample including only women that took HC. Most previous studies that assessed the effect of menstrual cycle phase on reward processing did not include such a control sample [e.g., Ref. (15)]. Accordingly, when the cycle group was considered alone in our study, the effect size for the interaction of “*phase*” × “*learning capacity*” × “*DAT1-genotype*” increased from 0.04 to 0.12 (see Table 1). Nevertheless, even the smaller effect size of 0.04 is in the range of effect sizes reported by pharmacological intervention studies published in the field. For example, none of the effect sizes reported by Eisenegger et al. (42) for interactions involving the factor “*DA intervention*” and “*DAT1-genotype*” in relation to social reward learning were higher than a partial eta squared of 0.098. When considering the fact that we assessed the impact of the physiological rise in E2 level, our findings indicate that endogenous E2 may be a quite potent modulator of DA transmission with effect sizes comparable to pharmacological agents like l-DOPA. In addition to that, like many of the previous studies on the role of the DAT1-polymorphism in reward processing, our study also did not control for other, potentially relevant genetic polymorphisms that could equally well affect reinforcement learning capacity. Future studies with bigger samples (*n* > 200) should use more advanced behavioral genetic methods like genome-wide association or at least haplotype analysis in order to draw a more comprehensive picture of cycle phase by genotype interactions relevant for inter- and intra-individual variations in reinforcement learning capacity.

Another limitation might be the use of a social feedback (smiley versus grumpy face) in the probabilistic feedback task. This was done to create a design that was comparable to Frank et al. (11), Klein et al. (12), and Diekhof and Ratnayake (9). However, a social feedback may have a lower salience than a monetary reward or loss and may thus lead to a reduced dopaminergic response, i.e., a less effective DA burst or dip, which could have made it easier for variations in E2 to actually tip the balance in favor of reward at the expense of punishment sensitivity. Even though, this thought is currently mere speculation, future studies have to address this potential confound and should test, whether learning from a monetary loss during action selection is equally affected by follicular E2.

Finally, the present study assessed the influence of the rise in endogenous E2 concentration, which precludes any solid inferences on causality. Therefore, placebo-controlled E2 administration studies in young women or a comparison between menopausal women who receive a hormone-replacement therapy or not will be important in that context [e.g., Ref. (58)]. Yet, pharmacological E2 may also have certain disadvantages like the possibility of supraphysiological stimulation in young women, as already outlined above, or the stimulation of a neural structure that might be already compromised by biological aging effects. As an initial step, the understanding of physiological E2 and its role in reinforcement learning may thus be crucial to provide an informed basis for future studies that use pharmacological intervention.

## Conclusion

Taken together, the present study adds to the growing awareness of the complex interplay between various physiological determinants of dopaminergic transmission. The observed effects on reinforcement learning capacity cannot simply be attributed to cycle phase or genotype alone, but may be a result of their interaction. Furthermore, the present data may provide preliminary evidence for a differential effect of natural and synthetic hormones on reinforcement learning capacity. In that way, they may not only point out the necessity to control for hormonal state and biological sex in behavioral genetics research, but may also offer new ideas for studies in clinical settings.

## Ethics Statement

This study was carried out in accordance with the recommendations of the local ethics committee “Ethikkommission der Ärztekammer Hamburg (Germany)” with written informed consent from all subjects. All subjects gave written informed consent in accordance with the Declaration of Helsinki. The protocol was approved by the “Ethikkommission der Ärztekammer Hamburg (Germany).”

## Author Contributions

ED designed the research, supervised data collection, analyzed the data, and wrote the paper. KJ collected the data, analyzed the data, and reviewed the manuscript. HE collected the data and reviewed the manuscript. SH supervised data collection and reviewed the manuscript. LR supervised data collection and reviewed the manuscript.

## Conflict of Interest Statement

The authors declare that the research was conducted in the absence of any commercial or financial relationships that could be construed as a potential conflict of interest.
